# Neoadjuvant chemotherapy for soft‐tissue sarcoma of the extremities: A post‐hoc Sarculator‐based risk analysis of the EORTC 62961–ESHO 95 randomized trial

**DOI:** 10.1002/cncr.70427

**Published:** 2026-04-26

**Authors:** Markus Albertsmeier, Valeria Milani, Lars H. Lindner, Gabriele Tinè, Sandro Pasquali, Dario Callegaro, Alessandro Gronchi, Hans‐Roland Dürr, Alexander Klein, Dorit Di Gioia, Sultan Abdel‐Rahman, Michael Schmidt, Jens Werner, Michael von Bergwelt‐Baildon, Rosalba Miceli, Rolf Issels

**Affiliations:** ^1^ Department of General Visceral and Transplantation Surgery LMU Hospital Ludwig‐Maximilians‐Universität Munich Munich Germany; ^2^ Department of Internal Medicine III LMU Hospital Ludwig‐Maximilians‐Universität Munich Munich Germany; ^3^ Zentrum für Integrative Onkologie Zürich Zürich Switzerland; ^4^ Biostatistics for Clinical Research Unit, Fondazione IRCCS Istituto Nazionale dei Tumori Milan Italy; ^5^ Department of Surgery Sarcoma Surgery Fondazione IRCCS Istituto Nazionale dei Tumori Milan Italy; ^6^ Department of Orthopedic Surgery, Physical Medicine, and Rehabilitation LMU Hospital Ludwig‐Maximilians‐Universität Munich Munich Germany; ^7^ Institute for Medical Information Processing, Biometry, and Epidemiology Ludwig‐Maximilians‐Universität Munich Munich Germany

**Keywords:** extremities, neoadjuvant chemotherapy, regional hyperthermia, risk stratification, Sarculator, soft tissue sarcoma, survival

## Abstract

**Background:**

In the EORTC 62961–ESHO 95 randomized trial (European Organization for Research and Treatment 62961–European Society of Hyperthermia Oncology 95; ClinicalTrials.gov identifier NCT00003052), neoadjuvant chemotherapy (NAC) combined with regional hyperthermia (RHT) improved survival in patients with soft tissue sarcoma (tumor size >5 cm, grade 2 or 3, deep location). This study investigated the survival benefit of NAC + RHT in a subgroup of patients who had extremity soft tissue sarcoma (ESTS) according to risk predictions using the Sarculator nomogram.

**Methods:**

Overall survival (OS) was predicted with the Sarculator nomogram using baseline prognostic parameters. Kaplan–Meier analysis was used to estimate observed OS. A bivariable Cox model including the Sarculator score, treatment, and their interaction was fitted. Hazard ratios for OS were calculated for each decile of the Sarculator risk distribution.

**Results:**

Of 143 patients with ESTS, 135 were analyzed (NAC, *n* = 70; NAC + RHT, *n* = 65) with a median follow‐up of 136 months (interquartile range, 110–183 months). Survival in the NAC + RHT group exceeded Sarculator predictions and improved compared with the group that received NAC alone (hazard ratio, 0.67; 95% confidence interval, 0.39–1.17; *p* = .081), with an absolute 5‐year OS difference of 15.6% (95% confidence interval, 0.0%–31.4%). Risk stratification suggested greater benefit of NAC + RHT as predicted OS decreased. However, the interaction between Sarculator score and treatment was not significant (*p* = .495).

**Conclusions:**

This analysis of ESTS from a randomized trial confirmed the previously reported OS benefit by adding RHT to NAC. Although patients with higher predicted risk seemed to benefit more from the combined treatment, these findings do not suggest that treatment decisions should be based on risk estimates alone, supporting the use of RHT combined with chemotherapy in patients who have primary ESTS.

## Abstract 2

In a Sarculator‐based subgroup analysis of patients with extremity soft tissue sarcoma patients from the EORTC 62961–ESHO 95 trial, the authors observed a benefit of regional hyperthermia in combination with neoadjuvant chemotherapy across the prognostic spectrum. These results support regional hyperthermia as part of neoadjuvant treatment for primary extremity soft tissue sarcoma.

## Plain Language Summary

- In a previous clinical trial, adding regional hyperthermia—targeted heating of the tumor area—to standard chemotherapy before surgery improved long‐term survival for patients with high‐risk soft tissue sarcomas.
- In this study, the authors re‐analyzed data from that trial using the Sarculator, a modern tool that predicts a patient’s individual risk.
- The authors observed that patients across all risk levels—but within the trial's high‐risk inclusion criteria—tended to live longer when hyperthermia was added to chemotherapy, with the greatest benefit seen in higher risk patients.
- These results support using hyperthermia as part of treatment for patients who have newly diagnosed extremity sarcomas.

## INTRODUCTION

Soft tissue sarcomas (STS) are associated with a significant risk of local recurrence and distant metastasis (DM), particularly in patients presenting with large, high‐grade tumors.^1^ Although radiotherapy has demonstrated improved local control in high‐risk extremity STS (ESTS),^2^ the clinical benefit of neoadjuvant chemotherapy (NAC) or adjuvant chemotherapy remains controversial.^3^, ^4^ The European Organization for Research and Treatment of Cancer (EORTC)‐Soft Tissue and Bone Sarcoma Group (EORTC‐STBSG) 62931 randomized trial—the largest adjuvant study of chemotherapy in STS—failed to demonstrate a survival advantage for adjuvant chemotherapy.^3^ Subsequently, the Italian Sarcoma Group (ISG)‐STS 1001 randomized trial (ClinicalTrials.gov identifier NCT01710176) compared neoadjuvant anthracycline‐ifosfamide (AI) chemotherapy with histotype‐tailored regimens in patients with ESTS and trunk wall sarcoma. The trial was terminated early after an interim analysis demonstrated superior survival with the standard AI regimen compared with histotype‐specific, anthracycline‐free regimens, except for trabectedin in patients who had myxoid liposarcoma.^4^

The heterogeneous results of these trials have prompted efforts to better identify which patients with ESTS are most likely to benefit from systemic therapy. One approach used prognostic nomograms post hoc to stratify patients according to their predicted survival probabilities.^5^, ^6^ The Sarculator is a well validated, widely used nomogram for ESTS^7^ that estimates overall survival (OS) and the crude cumulative incidence of DM (CCI‐DM) risk based on clinicopathologic variables. When applied to the EORTC‐STBSG 62931 and ISG‐STS 1001 studies, Sarculator identified subgroups of high‐risk patients with a predicted 10‐year OS rate <60% in both trials who appeared to benefit from chemotherapy, underscoring the potential utility of prognostic nomograms in guiding therapeutic decisions.

Independent of these later advances in prognostic stratification, alternative neoadjuvant treatment modalities have been explored to improve outcomes in high‐risk STS. One such strategy uses the combination of NAC with regional hyperthermia (RHT) in patients with high‐risk STS. Heat exposure (range, 40°C–43°C) of cancer cells in preclinical models and hyperthermia applied regionally to patients in early clinical studies have exhibited synergistic activity with ionizing radiation and chemotherapy.^8^ The safety and efficacy of combining RHT with NAC in patients with STS were demonstrated in the EORTC 62961–European Society of Hyperthermia Oncology (ESHO) 95 multicenter, randomized trial (ClinicalTrials.gov identifier NCT00003052),^9^ which compared neoadjuvant AI‐based chemotherapy combined with RHT versus NAC alone. High‐risk disease was defined by tumor size > 5 cm, French National Federation of Cancer Centers (FNCLCC) grade 2 or 3, and deep tumor location, reflecting contemporaneous risk criteria.^10^ Combined treatment significantly improved long‐term survival outcomes (hazard ratio [HR], 0.73; 95% confidence interval [CI], 0.54–0.98; *p* = .04).^11^

Although this trial remains the only large, randomized controlled study to demonstrate a positive effect of NAC in adult STS, the role of RHT has been controversial, in part because the trial used comparatively broad clinical risk definitions. Because treatment selection in modern sarcoma care increasingly relies on individualized prognostic models, it remains unclear whether the survival benefit observed with NAC + RHT persists across current risk strata or is confined to specific prognostic subgroups. Therefore, our objective was to describe the treatment effect of NAC + RHT compared with NAC alone in different Sarculator‐predicted risk groups of patients with ESTS in the EORTC 62961–ESHO 95 trial.

## MATERIALS AND METHODS

### Trial design and patients

The current study analyzed the subgroup of patients with ESTS enrolled in the EORTC 62961–ESHO 95 trial, an open‐label, multicenter, parallel‐group, phase 3 trial previously reported in detail.^10^, ^11^ Briefly, the trial included adults aged 18–70 years with histologically confirmed, high‐risk STS, defined by a greatest tumor dimension ≥5 cm, FNCLCC grade 2 or 3, or location deep to the fascia, and without DM. For patients who had undergone prior surgical resection with tumor‐free margins <1 cm, randomization was permitted within 8 weeks of surgery. Randomization was stratified according to tumor site (ESTS vs. non‐ESTS) and presentation (primary vs. recurrent). Patients were recruited at nine centers across Europe and North America (six from Germany) from July 1997 to November 2006 and were followed until December 2014.

Participants were to receive four cycles of NAC with combined doxorubicin, ifosfamide, and etoposide. In the experimental arm, NAC was combined with RHT (at 42°C for 60 minutes), which was administered concurrently with ifosfamide on days 1 and 4 of each chemotherapy cycle. Postoperatively, patients were to receive another four cycles of chemotherapy, either alone or in combination with RHT. The primary end point of the trial was local progression‐free survival, with secondary outcomes including survival and treatment‐related adverse events.

Because the Sarculator includes distinct models for ESTS^7^ and retroperitoneal STS,^12^ we separated the trial cohort by tumor location and the present analysis focused exclusively on patients with ESTS. To align the cohort with the parameters used by the Sarculator, patient inclusion was limited to specific histologic subtypes: dedifferentiated liposarcoma, leiomyosarcoma, synovial sarcoma, myxofibrosarcoma, myxoid liposarcoma, and undifferentiated pleomorphic sarcomas. Patients with Ewing sarcoma, rhabdomyosarcoma, or those treated for tumor recurrences were excluded. The original EORTC 62961–ESHO 95 trial was approved by local ethics committees at participating centers, and written informed consent was obtained from all participants. The current secondary analysis of anonymized data was approved with a waiver of additional review by the ethics committee at the medical faculty of Ludwig‐Maximilians University Munich (24‐0931).

### Statistical analysis

The primary end points of this analysis were OS and the incidence of DM. According to per the EORTC 62961–ESHO 95 trial protocol, OS was defined as the time until death resulting from sarcoma or its treatment, with survival times censored at the last follow‐up. In the original publication,^8^ deaths from other causes were not classified as events and were censored at the time of occurrence. To ensure consistency with the original trial data, the current analysis maintained this definition of OS. The median follow‐up was calculated using the reverse Kaplan–Meier method.

OS curves were estimated using the Kaplan–Meier method, and the CCI‐DM was estimated by accounting for competing events (i.e., local relapse, or death without DM, whichever occurred first). Kaplan–Meier and CCI‐DM curves were stratified by treatment arm. Because the original trial demonstrated a superior outcome with NAC + RHT versus NAC alone, OS between treatment groups was assessed using a one‐sided test. All other analyses were conducted using two‐sided tests, and statistical significance was defined as a *p* value < .05.

#### Sarculator's calibration and discriminative ability

We used the Harrell C‐index to quantify the Sarculator's discriminative ability to differentiate between patients at higher and lower risk.^13^ A C‐index of 0.5 indicates no better discrimination than would be expected by chance, whereas a value of 1.0 indicates perfect discrimination. Calibration was evaluated graphically through the construction of calibration curves, assessing the alignment between predicted and observed OS or CCI‐DM at 5 and 10 years. Patients were stratified into four equally sized subgroups based on their Sarculator‐predicted OS or CCI‐DM and were compared with the observed outcome (i.e., observed Kaplan–Meier OS and CCI‐DM).

### Sarculator as a predictive biomarker of the RHT effect

Sarculator is a tool that calculates OS and the CCI‐DM estimates at specific time points. These estimates are obtained using statistical models and are functions of a linear combination of covariate‐specific, model‐estimated coefficients and patient‐specific covariate values. This linear combination defines a time‐independent risk score. Higher scores correspond to lower OS or higher CCI‐DM, defining high‐risk populations.

To explore the Sarculator's role as a predictive biomarker for the effect of RHT on OS and CCI‐DM, we fitted a bivariate Cox model for OS and a Fine and Gray model^14^ for CCI‐DM, incorporating the Sarculator risk score, the treatment arm, and their interaction. The significance of this interaction term would indicate the value of the Sarculator as a predictive biomarker. A descriptive assessment of the interaction was performed by dividing the Sarculator risk score distribution into deciles and graphically displaying the model‐estimated relative risk measures (HRs from the Cox model, and subdistribution HRs [sHRs] from the Fine and Gray model) along with their corresponding 95% CIs. From these models, we also derived patient‐specific predicted OS and CCI‐DM curves, which were then averaged to obtain the overall Sarculator‐predicted curves.

In addition, we performed an OS analysis stratifying patients into higher and lower risk subgroups using the 60% cut‐off for Sarculator‐predicted 10‐year OS identified in prior analyses of the ISG/GEIS (Spanish Group for Research on Sarcoma) perioperative chemotherapy trial.^15^, ^16^ This threshold, corresponding to the median predicted OS in that trial, was previously validated in post‐hoc analyses of the EORTC 62931 adjuvant chemotherapy trial, in which chemotherapy benefit was confined to the higher risk group.^5^

## RESULTS

### Patients and treatment

**FIGURE 1:**
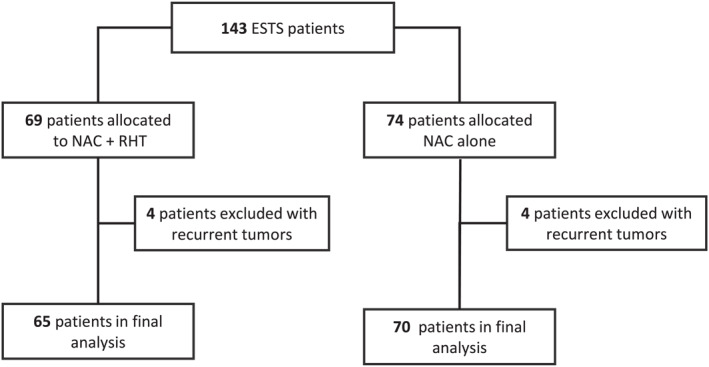
Flow chart of patient selection for the Sarculator‐based analysis. Of 143 patients with ESTS in the EORTC 62961–ESHO 95 randomized trial, 69 patients had been allocated to NAC combined with RHT, and 74 patients had been allocated to NAC alone. Four patients from each treatment arm were not eligible for the Sarculator‐based analysis because they had been enrolled in the trial with a recurrent tumor. EORTC indicates European Organization for Research and Treatment of Cancer; ESHO, European Society of Hyperthermia Oncology; ESTS, extremity soft tissue sarcoma; NAC, neoadjuvant chemotherapy; RHT, regional hyperthermia.

From the 341 patients in the EORTC 62961 trial, 143 patients with ESTS were randomized to receive either NAC alone or in combination with RHT, and 135 of these patients (NAC alone, *n* = 70; NAC + RHT, *n* = 65) were eligible for Sarculator analysis. Patient inclusion and exclusion are depicted in a flow chart (Figure 1), and the baseline characteristics of the final patient cohort are described in Table 1. Patients were followed for a median of 136 months (interquartile range, 110–183 months), allowing 5‐year and 10‐year OS and CCI‐DM predictions. By December 2014, 52 patients (39%) had died, and 83 patients (61%) were still alive. DM had occurred in 54 patients (40%), and 32 patients (24%) had experienced local relapse.

**TABLE 1:** Baseline characteristics of study patients compared with the Sarculator development cohort.

Variable	No. (%)
No. of patients	135 (100.0)
Age: Mean ± SD, years	49.1 ± 13.7
Tumor size: Mean ± SD, cm	12.3 ± 6.7
FNCLCC grade	
1	0 (0.0)
2	62 (45.9)
3	73 (54.1)
Histology	
UPS	33 (24.4)
Synovial sarcoma	22 (16.3)
DD/pleomorphic LPS	21 (15.6)
LMS	16 (11.9)
Myxofibrosarcoma	4 (3.0)
Myxoid LPS	1 (0.7)
WDLPS	0 (0.0)
MPNST	0 (0.0)
Other	38 (28.1)

Abbreviations: DD, dedifferentiated; FNCLCC grade, French National Federation of Cancer Centers grading system for soft tissue sarcomas (1, low grade; 2, intermediate grade; 3, high grade); LPS, liposarcoma; LMS, leiomyosarcoma; MPNST, malignant peripheral nerve sheath tumor; SD, standard deviation; UPS, undifferentiated pleomorphic sarcoma; WDLPS, well‐differentiated liposarcoma.

### Observed OS and CCI‐DM

**FIGURE 2:**
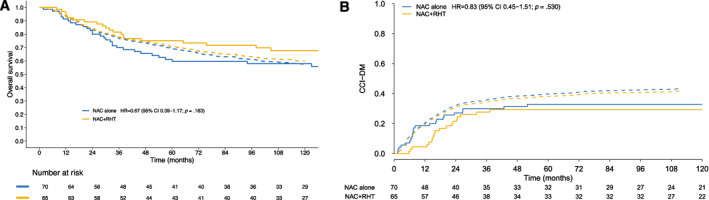
Predicted and observed OS and CCI‐DM stratified by treatment arm. (A) The average Sarculator predictions of OS (dashed lines) and the Kaplan–Meier estimates of observed OS (solid lines) stratified by treatment arm are illustrated. (B) The average Sarculator predictions of CCI‐DM (dashed lines) and the estimates of observed CCI‐DM (solid lines) stratified by treatment arm are illustrated. CCI‐DM indicates crude cumulative incidence of distant metastasis; CI, confidence interval; HR, hazard ratio; NAC, neoadjuvant chemotherapy; RHT, regional hyperthermia.

The results of the EORTC 62961–ESHO 95 trial have been published previously.^11^ Within the ESTS subcohort included in this study, the observed 5‐year OS was 59.6% for patients who received NAC alone and 75.2% for patients after NAC + RHT (absolute difference, 15.6%; 95% CI, 0.0%–31.4%), with 10‐year OS rates of 58.0% and 67.7%, respectively (Figure 2A, solid lines). Patients who received NAC + RHT achieved survival outcomes that surpassed Sarculator estimates, indicating a favorable effect relative to NAC alone (HR, 0.67; 95% CI, 0.39–1.17; one‐sided *p* = .081). The univariable Cox model HR of NAC + RHT versus NAC was 0.67 (*p* = .163; Table 2, univariable Cox model).

**TABLE 2:** Sarculator as a predictive biomarker of the regional hyperthermia effect on overall survival and the incidence of distant metastasis.^a^

	Overall survival			Distant metastasis		
	Univariable Cox model			Univariable Fine and Gray model		
Variable	HR	95% CI	*p*	sHR	95% CI	*P*
Treatment						
NAC alone	1.0			1.00	0.45–1.51	.530
NAC + RHT	0.67	0.39–1.17	.163	0.83	—	

	Overall survival			Distant metastasis		
	Bivariable Cox model			Bivariable Fine and Gray model		
Variable	HR	95% CI	*p*	sHR	95% CI	*P*
Treatment, Sarculator low‐risk^b^						
NAC alone	1.0			1.00		
NAC + RHT	0.83	0.37–1.84		1.30	0.51–3.32	
Treatment, Sarculator high‐risk^c^						
NAC alone	1.0			1.00		
NAC + RHT	0.64	0.36–1.14	.495^d^	0.65	0.34–1.23	.057^d^

Abbreviations: CI, confidence interval; HR, hazard ratio; NAC, neoadjuvant chemotherapy; OS, overall survival; RHT, regional hyperthermia; sHR, subdistribution hazard ratio.

^a^ Regression models for overall survival (A and B) and the cumulative incidence of disease‐specific mortality (C and D). A and C show univariable models with the treatment arm as the sole predictor; B and D show bivariable models that include treatment arm, Sarculator risk class, and their interaction term. HRs and sHRs refer to the comparison of NAC alone versus NAC + RHT.

^b^ Sarculator low‐risk indicates patients in the first quartile of the Sarculator distribution, corresponding to a predicted 10‐year overall survival rate >60%.

^c^ Sarculator high‐risk indicates patients in the third quartile of the Sarculator distribution.

^d^ These are *p* values for the interaction between treatment arm and Sarculator‐predicted risk.

The 5‐year and 10‐year CCI‐DMs were both 33% after NAC alone and 29% after NAC + RHT (Figure 2B, solid lines). Three metastatic events occurred after 3 years of follow‐up, including two events in the NAC‐alone group and one event in the NAC + RHT group, and none occurred after 5 years. In the univariable Fine and Gray model, the sHR was 0.83 (*p* = .530; Table 2, univariable Fine and Gray model).

#### Sarculator's calibration and discriminative ability

The Sarculator‐predicted OS and CCI‐DM curves in the two study arms overlapped in accordance with the randomized design, because the Sarculator covariates were equally distributed (Figure 2). In the NAC‐alone arm, Sarculator overestimated observed Kaplan–Meier OS, with convergence at 10 years, whereas it underestimated OS in the NAC + RHT arm. Sarculator overestimated the observed CCI‐DM in both treatment arms.

Calibration plots for OS (see Figure S1) and the CCI‐DM (see Figure S2) confirmed these patterns and illustrated differences across risk subgroups. Regarding 5‐year OS (see Figure S1A), Sarculator slightly overestimated the observed Kaplan–Meier estimates in the two higher risk subgroups (low OS) in the NAC‐alone arm, whereas the observed and predicted OS were more closely aligned in the two lower risk subgroups (high OS). At 10 years (see Figure S1C), the calibration was nearly perfect. In the NAC + RHT arm, for both 5‐year and 10‐year OS (Figure S1B,D, respectively), Sarculator largely underestimated the observed OS in the highest risk subgroup (lowest OS), suggesting a potential effect of RHT in patients with poor OS. The Harrell C‐index for OS predictions was 0.638 in the entire cohort, 0.632 in the NAC‐alone arm, and 0.638 in the NAC + RHT arm.

Sarculator slightly overestimated the CCI‐DM at 5 and 10 years in all subgroups (see Figure S2). The Harrell C‐index for DM predictions at 5 and 10 years was 0.721 and 0.640, respectively.

### Sarculator as predictive biomarker of the RHT effect

The results of the bivariable Cox model for OS and the bivariable Fine and Gray model for the CCI‐DM, both incorporating the interaction between the Sarculator risk score and treatment arms are shown in Table 2.

**FIGURE 3:**
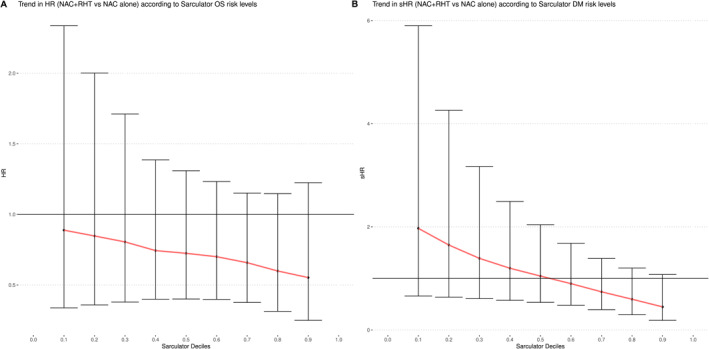
Treatment effect of regional hyperthermia in addition to neoadjuvant chemotherapy on OS and DM development at different Sarculator risk score deciles. (A) The treatment effect on OS was estimated as an HR from a bivariable model including the treatment arm, the Sarculator risk score, and their interaction. (B) For DM, the treatment effect was estimated as an sHR from a bivariable Fine and Gray model. DM indicates distant metastasis; HR, hazard ratio; NAC, neoadjuvant chemotherapy; OS, overall survival; RHT, regional hyperthermia; sHR, subdistribution hazard ratio.

In the Cox model of OS, the interaction term between treatment and the Sarculator risk score was not statistically significant (*p* = .495; Table 2, bivariable Cox model), indicating that, overall, the HR of NAC + RHT versus NAC alone did not vary significantly across Sarculator risk levels. However, because calibration results suggested that the addition of RHT might be more beneficial in patients identified by Sarculator as having a high risk of death, we calculated the HRs of OS for NAC + RHT versus NAC alone for each decile of the Sarculator risk score distribution (Figure 3A). A decreasing trend in treatment HRs was observed with increasing risk levels, suggesting that RHT may be more beneficial in higher risk patients.

**FIGURE 4:**
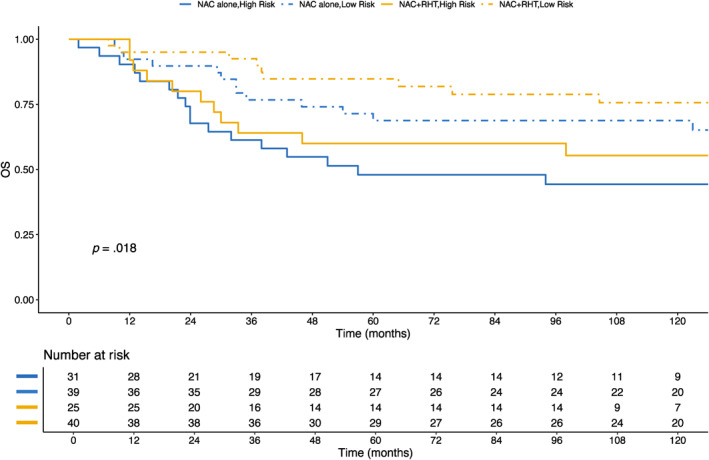
OS stratified by treatment arm and Sarculator risk class. Kaplan–Meier curves illustrate OS for patients treated with either NAC alone or NAC combined with RHT, stratified by Sarculator‐predicted 10‐year OS. Solid lines represent the high‐risk class (predicted OS <60%), and dashed lines represent the low‐risk class (predicted OS ≥60%). NAC indicates neoadjuvant chemotherapy; OS, overall survival; RHT, regional hyperthermia.

In addition, when patients were stratified into high‐risk and low‐risk groups according to the validated 10‐year 60% predicted OS cutoff, no significant association was observed between risk group and treatment effect (Figure 4). NAC + RHT appeared to be effective compared with NAC alone in both higher risk patients (HR, 0.80; 95% CI, 0.39–1.69) and lower risk patients (HR, 0.59; 95% CI, 0.26–1.36).

In the Fine and Gray model of the CCI‐DM, the interaction term between treatment and Sarculator risk score approached statistical significance (*p* = .057; Table 2, bivariable Fine and Gray model), suggesting that the treatment effect on DM might differ according to the risk levels determined by Sarculator, with RHT potentially offering greater benefit in higher risk individuals. This trend is illustrated in Figure 3B, which illustrates sHRs of the CCI‐DM for NAC + RHT versus NAC alone for each decile of the Sarculator risk score distribution.

## DISCUSSION

This study demonstrated that the survival benefit of NAC + RHT over NAC alone observed in the EORTC 62961–ESHO 95 randomized trial was maintained when outcomes of the ESTS subgroup were examined according to Sarculator predictions. Within the trial inclusion criteria (FNCLCC grade 2 or 3, tumor size ≥5 cm, location deep to the fascia), patients across different Sarculator‐defined risk categories, including those with a predicted 5‐year OS rate ≥60%, experienced improved survival after NAC + RHT compared with NAC alone.

Although previous studies pointed to a survival benefit from NAC^6^ or adjuvant chemotherapy^5^ that was limited to patients who had ESTS with a predicted OS rate <60%, our findings suggest that the addition of RHT extends the treatment effect of NAC beyond this highest risk subgroup. One explanation for the observed benefit from the combined treatment in patients with a predicted OS rate ≥60% may be related to the high proportion of grade 2 tumors (58%) in this subgroup. In general, grade 2 tumors are known to be less chemosensitive than grade 3 tumors.^17^ This was also observed in the EORTC 62931 trial, in which more than one third of tumors in the chemotherapy arm were grade 2.^3^ Therefore, the absence of a chemotherapy benefit in that study may partially reflect a dilution of the treatment effect caused by the predominance of less chemosensitive grade 2 tumors. In contrast, this reduced chemosensitivity was not apparent when NAC was combined with RHT in the EORTC 62961–ESHO 95 trial. Under the combined regimen, patients with grade 2 tumors demonstrated a greater survival benefit than those with grade 3 tumors.^11^ Taken together, these findings support the hypothesis that RHT may enhance chemosensitivity, thereby mitigating the typically lower responsiveness of grade 2 sarcomas to systemic therapy.

The plateau of the cumulative incidence curves indicates that DM occurred predominantly within the first 3–5 years, consistent with the pattern of early metastatic dissemination in ESTS.^18^ It is interesting to note that RHT appeared to delay DM development during the first year, although the CCI‐DM equalized thereafter. The delay in DM suggests an immunologic abscopal effect of RHT, supported by previously reported, treatment‐induced changes in the tumor microenvironment, which were significantly associated with local progression‐free and disease‐free survival.^19^ Another explanation for the observed effect of RHT may be related to improved local control in the context of a competing risk framework.

Prognostication of STS is challenging because of the diversity of prognostic factors, which is further compounded by the rarity of these tumors. Nomograms are helpful prognostic tools that assess multiple variables simultaneously, providing estimates of the individual likelihoods of specific outcomes at defined time points.^20^ To our knowledge, this is the first analysis to re‐assess a randomized neoadjuvant RHT trial using a contemporary, individualized risk model, thereby contextualizing its relevance for current clinical practice. In this regard, the use of trial data with a median follow‐up period >11 years is a strength of our study. Sarculator demonstrated acceptable discrimination and calibration in this cohort, supporting its prognostic validity in the context of multimodal therapy. Short‐term survival in patients with a poor prognosis may be overestimated, whereas long‐term survival predictions were more accurate.

For DM, the model appeared to slightly overpredict the risk at both 5 and 10 years, regardless of the treatment arm. Because only 26% of patients in the Sarculator development cohort received systemic treatment,^7^ compared with all patients in the EORTC 62961–ESHO 95 trial, and because systemic treatment is not a parameter in the Sarculator, the lower frequency of DM in the trial cohort may represent an effect of systemic treatment independent of RHT.

The previously proposed 60% cutoff, derived from chemotherapy‐treated cohorts,^5^ showed limited value as a predictive biomarker for RHT benefit. In the bivariable OS model (including the continuous Sarculator score, treatment, and their interaction), the estimates (Table 2) and the corresponding plots (Figure 3A) suggested a trend toward a stronger treatment effect among patients with higher Sarculator scores (upper deciles), consistent with prior findings in chemotherapy‐treated cohorts. In contrast, Kaplan–Meier curves stratified by risk class (Figure 4) appeared to show similar benefits in both the low‐risk and high‐risk groups. This divergence likely reflects that the 60% cutoff is treatment‐specific and, in this cohort, does not capture RHT‐specific effects. As illustrated in Figure S1B, the Sarculator nomogram was not well calibrated for patients in the NAC + RHT arm who were at higher risk (predicted OS, <60%). This offers an alternative presentation of the finding illustrated in Figure 3A, expressed as observed versus predicted OS rather than as HRs. Therefore, our analysis demonstrates that, although the Sarculator provides robust prognostic information for patients with ESTS, it does not predict individual responses to RHT.

Two limitations regarding definitions in the EORTC trial need to be mentioned. First, the EORTC trial used a disease‐specific definition of survival, whereas Sarculator was calibrated for OS, with death from any cause counted as an event. This difference may not have had a strong influence on risk stratification, but it may have contributed to the underestimation of survival. Second, the Sarculator uses the date of surgery as the starting point for observation; whereas, in the EORTC 62961–ESHO 95 cohort, the starting point was the date of randomization. This variation may introduce a minor bias in the estimated probabilities but is unlikely to significantly affect overall results.

Finally, the relatively small sample size of the ESTS subgroup, particularly in the stratified analyses, limits the generalizability of our findings. Although this analysis provides additional insight into the role of RHT as part of multimodal therapy for ESTS, it should be interpreted cautiously. Because the trial eligibility criteria selected an intermediate‐risk to high‐risk population, the observed benefit in patients with a predicted OS rate ≥60% should not be extrapolated to those with truly low‐risk disease.

Within these eligibility boundaries, our findings indicate that the addition of RHT expands the therapeutic applicability of NAC. Specifically, RHT appears to permit the use of NAC in patients who have a comparatively favorable baseline prognosis for whom chemotherapy alone would not typically be considered. In this context, risk cutoffs derived from chemotherapy‐only cohorts may not be transferable to multimodal strategies that incorporate RHT. Further research is needed to identify predictive biomarkers for the response to RHT and to optimize patient selection for this treatment approach.

## CONCLUSION

In this Sarculator‐based analysis of the EORTC 62961–ESHO 95 trial, the addition of RHT to NAC was associated with improved OS across the prognostic spectrum, with a tendency toward greater benefit in patients with poorer baseline prognosis. Sarculator remained a strong prognostic tool but did not identify subgroups unlikely to benefit from RHT. These results further support the use of hyperthermia in the setting of NAC for patients with primary ESTS.

## AUTHOR CONTRIBUTIONS

**Markus Albertsmeier**: Conceptualization; data curation; methodology; investigation; visualization; project administration; writing—original draft. **Valeria Milani**: Conceptualization; data curation; investigation; writing—original draft; project administration. **Lars H. Lindner**: Conceptualization; funding acquisition; investigation; supervision; writing—review and editing. **Gabriele Tiné**: Methodology; investigation; formal analysis; visualization; writing—original draft; software. **Sandro Pasquali**: Conceptualization; methodology; writing—review and editing. **Dario Callegaro**: Methodology; writing—review and editing. **Alessandro Gronchi**: Conceptualization; writing—review and editing; supervision; resources. **Hans‐Roland Dürr**: Writing—review and editing; supervision. **Alexander Klein**: Validation; writing—review and editing. **Dorit Di Gioia**: Data curation; writing—review and editing. **Sultan Abdel‐Rahman**: Data curation; project administration; investigation; writing—review and editing; validation. **Michael Schmidt**: Methodology; formal analysis; data curation; validation; writing—review and editing. **Jens Werner**: Supervision; resources; writing—review and editing. **Michael von Bergwelt‐Baildon**: Supervision; resources; writing—review and editing. **Rosalba Miceli**: Conceptualization; methodology; formal analysis; visualization; writing—original draft; software; resources. **Rolf Issels**: Conceptualization; funding acquisition; investigation; supervision; writing—original draft; resources.

## CONFLICT OF INTEREST STATEMENT

Lars H. Lindner owns stock in Thermsome GmbH outside the submitted work. Sultan Abdel‐Rahman reports travel reimbursement from Dr Sennewald Medizintechnik GmbH outside the submitted work. Rosalba Miceli reports personal/consulting fees from Boehringer Ingelheim outside the submitted work. The remaining authors disclosed no conflicts of interest.

## Supporting information

Supplementary Material S1

Figure S1

Figure S2

## Data Availability

The data that support the findings of this study are available on request from the corresponding author. The data are not publicly available because of privacy or ethical restrictions.
