# Supplementary material for: Neoadjuvant chemotherapy for soft‐tissue sarcoma of the extremities: A post‐hoc Sarculator‐based risk analysis of the EORTC 62961–ESHO 95 randomized trial
Source: Cancer. 2026 Apr 26;132:e70427. doi: 10.1002/cncr.70427 (PMC13110853; doi:10.1002/cncr.70427)
Supplement: Supplementary file 1 — Supplementary Material S1 [file CNCR-132-e70427-s002.docx]

**FIGURE S1.** Calibration plots comparing Sarculator-predicted and observed overall survival (OS). Panels A and C show calibration in the neoadjuvant chemotherapy (NAC) alone arm at 5 and 10 years. Panels B and D show calibration in the NAC + regional hyperthermia (RHT) at 5 and 10 years. The Sarculator-predicted OS probabilities were stratified into four equally sized subgroups. Hence, for each subgroup, the average predicted probability (x-axis) was plotted against the Kaplan-Meier OS observed probability (y-axis). The 95% confidence intervals of the Kaplan-Meier estimates are represented as vertical lines.

**FIGURE S2.** Calibration plots comparing Sarculator-predicted and observed incidence of distant metastasis (CCI-DM). Panels A and C show calibration in the neoadjuvant chemotherapy (NAC) alone arm at 5 and 10 years. Panels B and D show calibration in the NAC + regional hyperthermia (RHT) at 5 and 10 years. The Sarculator-predicted DM probabilities were stratified into four equally sized subgroups. Hence, for each subgroup, the average predicted probability (x-axis) was plotted against the observed CCI-DM (y-axis). The 95% confidence intervals of the CCI-DM estimates are represented with vertical lines.
