# Supplementary figures and images for: Neoadjuvant chemotherapy for soft‐tissue sarcoma of the extremities: A post‐hoc Sarculator‐based risk analysis of the EORTC 62961–ESHO 95 randomized trial
Source: Cancer. 2026 Apr 26;132:e70427. doi: 10.1002/cncr.70427 (PMC13110853; doi:10.1002/cncr.70427)

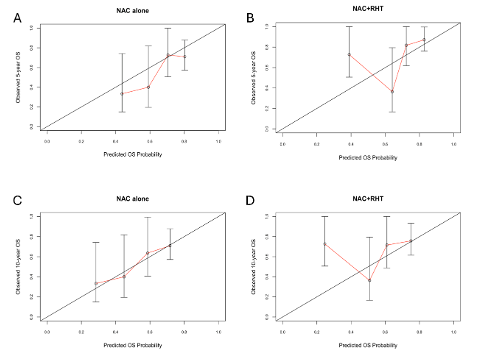

Supplement: Supplementary file 2 — Figure S1 [file CNCR-132-e70427-s001.tiff]

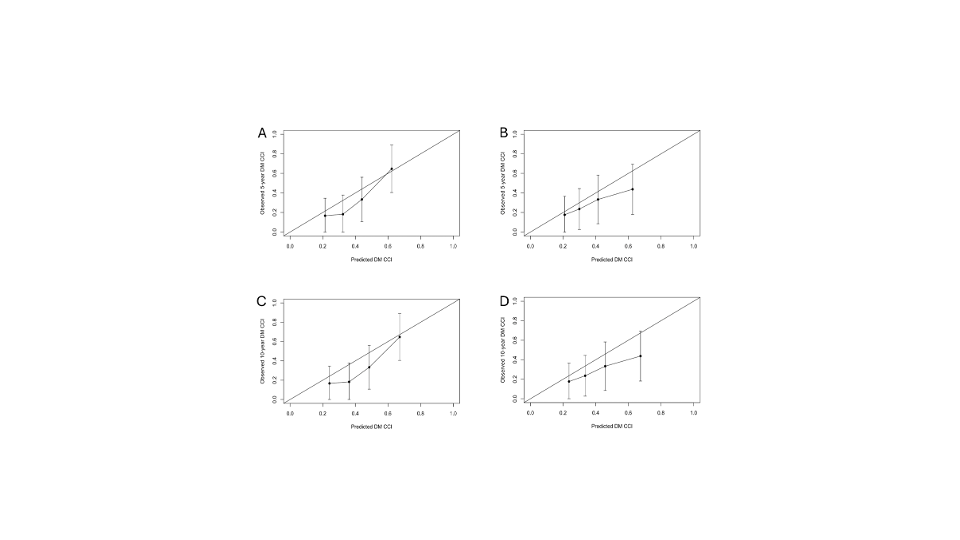

Supplement: Supplementary file 3 — Figure S2 [file CNCR-132-e70427-s003.tiff]
